# 人肺腺癌辐射耐受细胞株的建立及其放射抵抗机制探讨

**DOI:** 10.3779/j.issn.1009-3419.2023.102.08

**Published:** 2023-02-20

**Authors:** Jingjing ZHANG, Shenglin MA, Qiong WU

**Affiliations:** ^1^310006 杭州，浙江大学医学院附属杭州市第一人民医院转化医学研究中心，浙江大学癌症中心，浙江省临床肿瘤药理与毒理学研究重点实验室（张静静）; ^1^Department of Transitional Medicine Research Center, Key Laboratory of Clinical Cancer Pharmacology and Toxicology Research of Zhejiang Province, Zhejiang University Cancer Center, Affiliated Hangzhou First People’s Hospital, Zhejiang University School of Medicine, Hangzhou 310006, China; ^2^310002 杭州，浙江大学医学院附属杭州市肿瘤医院胸部肿瘤科（马胜林）; ^2^Department of Thoracic Oncology, Affiliated Hangzhou Cancer Hospital, Zhejiang University School of Medicine, Hangzhou 310002, China; ^3^310022 杭州，浙江省肿瘤医院，浙江省中医药重点实验室 中西医结合肿瘤学实验室（吴琼）; ^3^Integrated Traditional Chinese and Western Medicine Oncology Laboratory, Key Laboratory of Traditional Chinese Medicine of Zhejiang Province, Zhejiang Cancer Hospital, Hangzhou 310022, China

**Keywords:** 肺肿瘤, DNA损伤修复, 辐射耐受, Lung neoplasms, DNA damage repair, Radioresistance

## Abstract

**背景与目的:**

放疗是肺癌最常见的治疗方法之一，40%-50%的患者在放疗后会出现局部肿瘤未控或复发，放射抵抗是导致肺癌局部控制失败的重要原因。然而，体外放疗抵抗模型的缺乏是阻碍其机制研究的主要因素。因此，本研究旨在通过建立人肺腺癌H1975和H1299辐射耐受细胞株，为未来开展肺癌放射抵抗相关性研究创建体外模型，并初步探索其放射抵抗机制。

**方法:**

对H1975和H1299细胞进行等剂量的X射线分次照射，构建辐射耐受细胞株H1975DR和H1299DR；采用克隆形成实验比较H1975和H1975DR细胞、H1299和H1299DR细胞的克隆形成能力，线性二次模型拟合细胞存活分数曲线；DNA损伤修复能力的检测采用彗星实验，并计算DNA尾部百分比（Tail DNA%）；利用光学显微镜、CCK-8、FACS、细胞划痕和侵袭等方法比较细胞形态、细胞增殖能力、细胞凋亡水平和周期分布、细胞迁移和侵袭能力等生物学特性；通过Western blot免疫印迹分析DNA-PKcs、53BP1、RAD51、p-ATM等DNA损伤修复因子的表达。

**结果:**

历经5个月的照射及持续培养获得稳定的辐射耐受细胞株H1975DR和H1299DR。X射线照射下，两株辐射耐受株的细胞增殖活性、克隆形成能力、DNA损伤修复能力均显著提高；细胞周期G_2_期/M期比例均显著下调，G_0_期/G_1_期比例上调；细胞迁移及侵袭能力显著增强。非同源末端连接（nonhomologous end-joining, NHEJ）修复通路的p-DNA-PKcs（Ser2056）、53BP1及同源重组（homologous recombination, HR）修复通路的p-ATM（Ser1981）、RAD51相对蛋白表达量较H1975及H1299均有不同程度的增高。

**结论:**

通过等剂量分次照射可诱导H1975和H1299细胞株分化为肺腺癌辐射耐受细胞株H1975DR和H1299DR，为肺癌患者放疗抵抗的机制研究提供了体外细胞学模型。

## Abstract (en)

**Background and objective:**

Radiotherapy is one of the most common treatments for lung cancer, and about 40%-50% of patients after radiotherapy will appear uncontrolled or recurrence in the case of local tumors. Radioresistance is the predominant cause of local therapeutic failure. Nevertheless, the lack of in vitro radioresistance models is an influential factor obstructing the study of its mechanism. Therefore, the establishment of radioresistant cell lines, H1975DR and H1299DR, was beneficial to explore the mechanism of radioresistance in lung adenocarcinoma.

**Methods:**

The radioresistant cell lines of H1975DR and H1299DR were obtained from H1975 and H1299 cells irradiated with equal doses of X-rays; Clonogenic assays were performed to compare the clone-forming ability of H1975 vs H1975DR cells, H1299 vs H1299DR cells, then fitting cell survival curve by linear quadratic model; The comet assay was employed to examine DNA damage repair and calculate the percentage of DNA tails; The optical microscopy, CCK-8, flow cytometry, Transwell invasion assays were used to compare biological characteristics such as cell morphology, cell proliferation, apoptosis level, cycle distribution, cell migration and invasion; Western blot was carried out to measure the protein expression of DNA damage repair factors, such as DNA-PKcs, 53BP1, RAD51, and p-ATM.

**Results:**

After five months of continuous irradiation and stable culture, radioresistant cell lines H1975DR and H1299DR were obtained. The cell proliferation activity, clone formation ability and DNA damage repair ability of the two radioresistant cell lines were significantly improved under X-ray irradiation. The proportion of the G_2_/M phase was markedly decreased and the proportion of the G_0_/G_1_ phase was increased. Cell migration and invasion ability were significantly enhanced. Relative expression levels of p-DNA-PKcs (Ser2056), 53BP1 in the nonhomologous end-joining (NHEJ) repair pathway and p-ATM (Ser1981), RAD51 in the homologous recombination (HR) repair pathway were higher than those in H1975 and H1299.

**Conclusion:**

H1975 and H1299 cell lines can be able to differentiate into lung adenocarcinoma radioresistant cell lines H1975DR and H1299DR by equal dose fractional irradiation, which provided an in vitro cytological model for the study of radiotherapy resistance mechanism of lung cancer patients.

目前，肺癌作为全世界发病率（11.4%）和死亡率（18%）最高的癌症之一，它的总体预后较差，5年生存率低于20%^[1]^。非小细胞肺癌（non-small cell lung cancer, NSCLC）是最常见的原发性肺癌类型，占原发性肺癌病例的80%-85%以上。手术切除主要用于I期/II期/IIIA期NSCLC的治疗，对于不可切除的局部晚期及晚期NSCLC患者，化疗、靶向治疗、免疫治疗和精准放疗等是主要的肿瘤治疗方法^[2,3]^。在病程的不同阶段，60%-70%的肺癌患者需要进行放疗，然而放疗通常伴随着癌细胞不可避免地对射线产生抵抗，导致肿瘤的局部未控、复发和远处转移^[4,5]^。通过构建肺腺癌辐射耐受细胞模型及探索放射抵抗的发生机制，对寻求新的治疗策略用于辅助进展期NSCLC的放疗以提高肺癌放疗的局部控制率至关重要。

肿瘤细胞放射抵抗的发生是一个非常复杂的过程，涉及多种基因、多种因素和多种机制的共同调节。研究^[6]^显示DNA损伤修复、放疗诱导的促血管生成效应、细胞周期再分布及自噬调节等是肿瘤细胞放射抵抗的重要研究表型，其中肿瘤细胞DNA损伤修复能力增强是发生放射抵抗的关键机制。

DNA双链断裂（DNA double-strand breaks, DSB）被认为是最致命的DNA损伤形式。DSB可由新陈代谢发生氧化或烷化产生，或由DNA复制叉的终止内源性产生，或由摄入化学性物质和电离辐射（ionizing radiation, IR）外源性产生^[7]^。未修复或未正确修复的DSB会导致基因组不稳定从而诱发基因突变、神经变性、细胞衰老、免疫缺陷、细胞死亡或癌症^[8]^。哺乳动物细胞主要通过非同源末端连接（nonhomologous end-joining, NHEJ）和同源重组（homologous recombination, HR）来应答IR诱导的DSBs。NHEJ即在缺失同源性DNA序列的情况下对两个DNA的DSB末端进行连接，是一个快速且容易出错的过程，常常致使修复断裂部位出现小的缺失或插入。但由于该修复途径在整个细胞周期中处于活跃状态，且基因的编码或调控区仅占据染色体DNA的很小一部分，绝大多数可能是沉默的和/或非必要的。因此，虽然NHEJ是“快速、不规范”的，但这种修复途径可以为细胞争取最大的生存机会^[9]^。相较之，HR则是以同源未损伤的姐妹染色单体为模板精准地修复DSB，主要发生在S期/G_2_期，两种修复方式相辅相成，共同参与DSB全过程^[10]^。然而肿瘤放射抵抗的详细机制仍不明确，本研究采用等剂量分割放疗的方式构建了H1975DR和H1299DR肺腺癌辐射耐受细胞株并对其生物学特性进行探索，为肺癌患者放疗耐受的发生发展机制研究提供了体外细胞学模型。

## 1 材料与方法

### 1.1 细胞和主要试剂

人肺腺癌细胞H1975和H1299购自中国科学院上海细胞库。RPMI-1640培养基购自美国Gibco公司；胎牛血清购自德国Serana公司；青霉素和链霉素购自杭州科易生物技术有限公司；结晶紫购自美国Sigma公司；彗星检测试剂盒购自美国Trevigen公司；SYBR Green I荧光染色试剂购自美国Invitrogen公司；CCK-8（Cell Counting Kit-8）试剂盒购自美国MedChemExpress生物科技公司；FITC Annexin V细胞凋亡检测试剂盒、BD Pharmingen PI/RNase染色试剂和Matrigel均购自美国B&D公司；BCA蛋白浓度测定试剂盒购自武汉博士德生物工程有限公司；p-ATM（No.13050, 1:1,000）、ATM（No.2873, 1:1,000）、RAD51（No.8875, 1:1,000）、p-DNA-PKcs（No.68716, 1:1,000）、DNA-PKcs（No.38168, 1:1,000）、53BP1（No.4937, 1:1,000）、KU80（No.2753, 1:1,000）、p-p95/NBS1（No.3001, 1:1,000）、p95/NBS1（No.14956, 1:1,000）、β-actin（No.4970, 1:5,000）抗体、山羊抗兔/鼠HRP二抗均购自美国Cell Signaling Technology公司；p53（No.sc-126, 1:1000）、KU70（No.sc-17789, 1:1,000）抗体均购自上海Santa Cruz公司。

### 1.2 细胞培养

人肺腺癌细胞株H1975和H1299细胞用RPMI-1640培养基在5%CO_2_、37 ^o^C恒温培养箱中进行培养。

### 1.3 肺腺癌辐射耐受细胞株照射条件及方法

如图1A所示，取指数生长期H1975和H1299细胞（4×10^5^个/细胞）接种于T25培养瓶中，待细胞贴壁、融合度达60%-70%时，采用美国Precision X-ray Brand: X-RAD 225小动物辐照仪给予4 Gy X线照射。期间每2天更换新鲜培养液，当细胞密度达90%时消化传代，24 h后重复照射4 Gy。累积照射剂量达到60 Gy时（共经15次、4个月照射）停止照射并继续传代培养2代-3代，即为人肺腺癌辐射耐受细胞株H1299DR、H1975DR。在此过程中，亲代细胞不照射但同步培养传代。

**图1:**
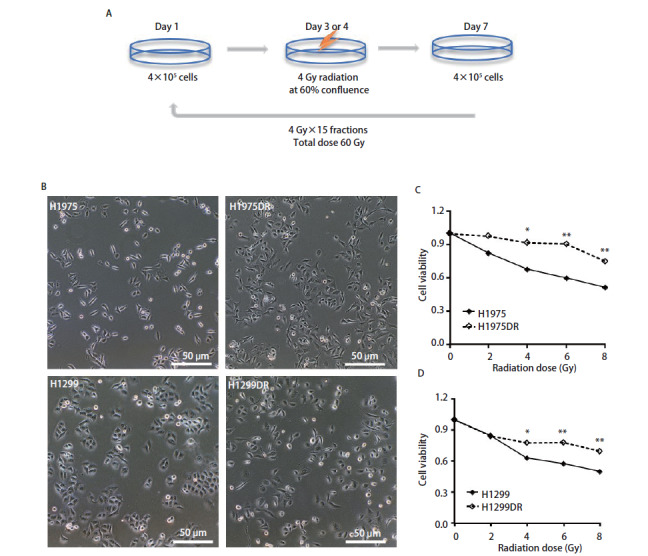
肺腺癌辐射耐受细胞株H1975DR和H1299DR增殖能力增强。 A：肺腺癌辐射耐受细胞株H1975DR和H1299DR构建模式图；B：H1975/H1975DR和H1299/H1299DR细胞形态光镜观察（×100）；C、D：H1975/H1975DR和H1299/H1299DR细胞生存率，分别给予单次剂量0 Gy、2 Gy、4 Gy、6 Gy、8 Gy照射72 h后CCK-8检测细胞增殖率。*P<0.05；**P<0.01。

### 1.4 CCK-8法检测细胞增殖能力

在96孔板中，以每孔3,000个/100 μL密度接种细胞，待细胞贴壁24 h后，亲本株和耐受株分别给予0 Gy、2 Gy、4 Gy、6 Gy和8 Gy照射，72 h后， 对450 nm波长处的吸光度（optical density, OD）进行测量。并计算细胞存活率：存活率（%）= 实验组OD_450_/对照组OD_450_。

### 1.5 克隆形成实验

取对数生长期的亲本株（H1975和H1299）和辐射耐受株（H1975DR和H1299DR），消化计数后铺板，分别给予0 Gy、2 Gy、4 Gy、6 Gy、8 Gy剂量照射。静置培养14 d，0.01%结晶紫染色30 min，计算集落数（≥50个细胞的集落）。运用线性二次模型SF=exp(-(α*D+β*(D^2)))拟合存活曲线。

### 1.6 彗星实验

细胞DNA的损伤程度用彗星检测试剂盒进行测定，单细胞凝胶电泳后，用SYBR Green I染色30 min，封片拍照。采用Comet Assay Software Pect（CASP）图像分析软件测量细胞尾部DNA含量（Tail DNA%）。

### 1.7 细胞凋亡和细胞周期实验

使用FITC Annexin V细胞凋亡检测试剂盒I进行细胞凋亡分析。使用BD Pharmingen PI/RNase染色试剂评估细胞周期。在RPMI-1640无血清培养基中同步化24 h，之后更换正常培养基继续培养24 h，比较两组细胞同步化前后细胞周期的变化。给予单次剂量6 Gy放射处理24 h、48 h、72 h后，收集1×10^6^个细胞并用预冷的75%乙醇在-20 °C下固定24 h，然后用PBS洗涤2次，并用PI/RNase染色缓冲液染色15 min。使用BD FACS Canto II对细胞周期和细胞凋亡样品进行流式细胞术分析。

### 1.8 细胞划痕实验

取100×10^4^个细胞/每孔均匀铺板于6孔板中，贴壁后，用灭菌的20 μL枪头，垂直划痕，用预热的PBS轻柔冲洗细胞，去除漂浮的细胞及细胞碎片，加入含1%FBS的RPMI-1640培养基，按0 h、12 h、24 h、30 h观察细胞的爬行状态，一般取横线上下相同的位置，当亲本株与辐射耐受株爬行面积出现显著差异时拍摄记录。

### 1.9 细胞侵袭实验

提前24 h在4 °C溶解Matrigel，并更换培养基为含0.2%BSA的无血清RPMI-1640培养基。按1:4比例稀释Matrigel，取50 μL混合液铺于上室内，置于37 °C培养箱中凝固2 h。上室加入无血清RPMI-1640培养基的细胞悬液，下室加入800 μL含10%FBS的RPMI-1640培养基。H1299/H1299DR及H1975/H1975DR细胞株分别于30 h与24 h后，用甲醇固定，0.01%结晶紫染色30 min，拍照并计数。

### 1.10 Western blot检测DNA损伤相关蛋白表达

取对数生长期的细胞接种于6孔板中，贴壁后，给予4 Gy放疗，24 h收取总蛋白并定量蛋白质浓度。采用十二烷基硫酸钠-聚丙烯酰胺凝胶电泳分离蛋白，再转移到PVDF膜上。5%脱脂牛奶封闭1 h后，将膜与一抗在4 °C下孵育过夜。

### 1.11 统计学分析

采用SPSS 26.0和GraphPad Prism 8.0进行数据分析及作图。采用t检验进行组间比较。P<0.05定义为具有统计学差异。

## 2 结果

### 2.1 肺腺癌辐射耐受株细胞增殖能力增强

如图1B所示，H1975/H1975DR细胞和H1299/H1299DR细胞呈单层贴壁生长，其中H1975细胞株呈细长梭形，膜边界清晰，折光性强，而H1975DR细胞株细胞体积增大，膜边界欠清晰，折光性较弱，细胞内的颗粒和空泡增多，可见多核；H1299细胞呈聚集性生长，形态较规则，边界清晰，而H1299DR细胞株细胞间隙明显增大，边界欠清晰，形态呈纺锤样演变，多长伪足。细胞生存率反映了H1975/H1975DR和H1299/H1299DR细胞株在不同剂量照射下细胞的增殖能力。如图1C和图1D所示，细胞在4 Gy、6 Gy和8 Gy剂量照射72 h后表现出生存率的差异（P<0.05），表现为与亲本株H1975及H1299相比，辐射耐受株H1975DR和H1299DR的总体生存曲线下降速度均变慢，表明X射线照射后72 h，辐射耐受株较亲本株细胞生存能力增强。

### 2.2 肺腺癌辐射耐受细胞株DNA损伤修复能力增强

采用克隆形成实验检测H1975DR和H1299DR细胞株的放射抵抗性，结果如图2A所示，随着放射剂量的递增（0 Gy、2 Gy、4 Gy、6 Gy、8 Gy），H1975/H1975DR及H1299/H1299DR细胞集落形成数均递减，说明放射剂量越大，对细胞的杀伤越大。且H1975DR和H1299DR细胞在接受较高剂量照射时，细胞集落形成能力明显较亲本株增强，具体表现为H1975/H1975DR在4 Gy、6 Gy、8 Gy，H1299/H1299DR在6 Gy、8 Gy照射时，H1975DR和H1299DR细胞克隆形成数均显著较多（图2A，P<0.05），细胞存活分数均增加（图2B，图2C，P<0.01），以上结果表明H1975DR和H1299DR细胞放射抗性增强，具有较强的克隆形成能力。同时，我们采用彗星实验直接检测损伤的DNA片段。结果如图2D、图2E所示，在给予单次剂量4 Gy照射1 h后，H1975/H1975DR及H1299/H1299DR细胞的Tail DNA%均显著增加，呈“彗星状”拖尾现象。8 h后结果显示，H1975DR和H1299DR细胞的Tail DNA%均明显低于肺腺癌亲本株（P<0.001），提示肺腺癌辐射耐受细胞株的DNA损伤修复能力显著增强，对射线的敏感性降低。

**图2:**
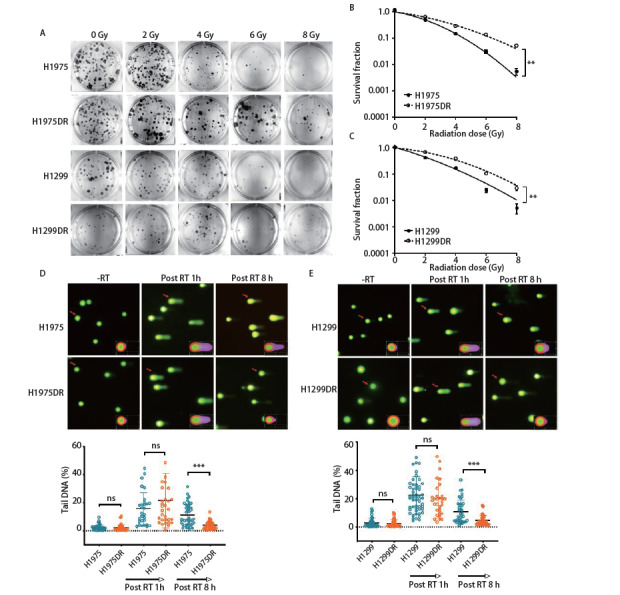
肺腺癌辐射耐受细胞株H1975DR和H1299DR DNA损伤修复能力增强。 A：H1975DR和H1299DR辐射耐受细胞株克隆形成能力增强。分别给予0 Gy、2 Gy、4 Gy、6 Gy、8 Gy单次剂量照射后计算克隆形成数，n=3孔/组；B、C：采用线性二次模型拟合H1975DR和H1299DR细胞剂量存活曲线；D、E：采用彗星实验观察放射对H1975/H1975DR和H1299/H1299DR细胞DNA损伤修复能力的影响。4 Gy照射1 h、8 h后进行单细胞凝胶电泳。下方为细胞彗星尾部DNA百分比的定量分析。每组至少检测50个细胞。**P<0.01；***P<0.001；ns: 无显著差异。

### 2.3 肺腺癌辐射耐受细胞株凋亡水平的改变

采用流式细胞仪检测亲本株H1975、H1299及辐射耐受株H1975DR、H1299DR放射前后细胞凋亡分布情况。如图3A、图3D所示，在基础状态下及6 Gy照射24 h后，亲本株与辐射耐受株的总凋亡率相近，无统计学差异。值得注意的是，在6 Gy照射48 h后，H1975DR细胞株的总凋亡率显著低于H1975细胞株（P<0.05）（图3B），表明H1975DR的放射抵抗性可能与细胞凋亡相关。

**图3:**
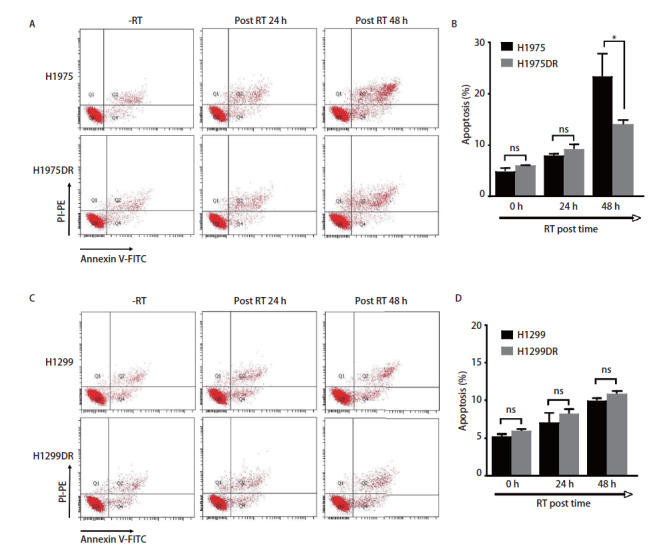
肺腺癌辐射耐受细胞株凋亡水平的改变。 采用流式细胞仪检测放射对H1975/H1975DR（A）和H1299/H1299DR（C）细胞凋亡水平的影响，B、D为凋亡占比统计图。*P<0.05。

### 2.4 肺腺癌辐射耐受细胞株细胞周期的再分布

采用流式细胞仪检测亲本株与辐射耐受株细胞周期分布的变化。H1975和H1975DR细胞在无血清培养基中同步化24 h后，H1975细胞周期G_0_期/G_1_期、S期、G_2_期/M期的比例分别为80.95%、7.75%、11.04%，H1975DR分别为70.86%、14.49%、14.66%，二者细胞均大部分被阻滞在G_0_期/G_1_期（图4A）；在含10%的胎牛血清培养基中去同步化24 h后，H1975与H1975DR细胞周期所占的比例较前均无明显变化，G_0_期/G_1_期分别为84.71%、71.47%，S期分别为7.11%、14.69%，G_2_期/M期分别为8.18%、13.84%。一致的是，H1299和H1299DR细胞在同步化24 h后，大部分细胞同样被阻滞在G_0_期/G_1_期，分别为77.08%、69.58%（图4B）；去同步化24 h后，H1299和H1299DR细胞G_0_期/G_1_期均明显下降，分别为55.19%、55.06%，S期和G_2_期/M期均分别大幅上升，分别为30.62%、25.79%和14.20%、19.16%，但去同步化后，H1299细胞与H1299DR细胞各周期所占的比例均无明显变化。以上结果表明，在基础状态下，辐射耐受株与亲本株细胞周期分布均无显著差异。

**图4:**
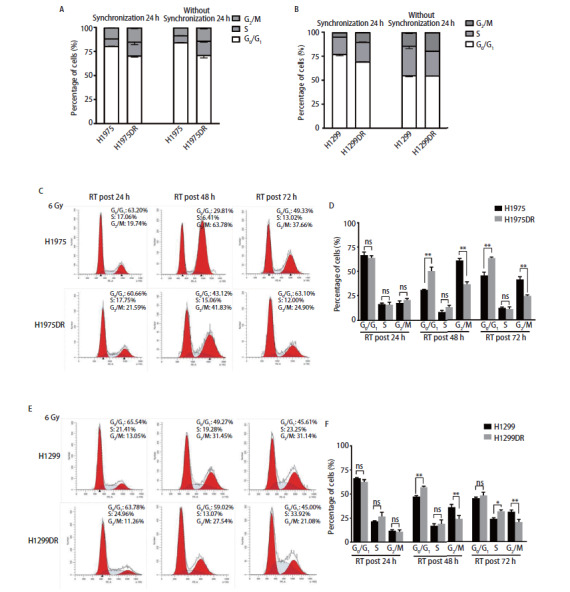
肺腺癌辐射耐受细胞株细胞周期的再分布。 A、B：H1975/H1975DR和H1299/H1299DR同步化前后周期分布；C-F：采用流式细胞术检测H1975DR（C、D）和H1299DR（E、F）辐射耐受细胞株单次剂量照射6 Gy后24 h、48 h、72 h后细胞周期的改变，D和F是细胞周期统计图。*P<0.05; **P<0.01。

X射线可诱导辐射耐受株H1975DR和H1299DR细胞发生细胞周期再分布。如图4C-图4F所示，给予单次剂量6 Gy照射后48 h，辐射耐受株H1975DR和H1299DR细胞的G_2_期/M期分布比例较亲本株H1975和H1299均显著下调（P<0.01），其中H1975和H1299细胞G_2_期/M期占比分别为63.78%和31.45%，H1975DR和H1299DR细胞占比减少，分别为41.83%、27.54%。同时，放射相对抵抗的G_0_期/G_1_期细胞周期分布比例均显著上调（P<0.05），其中H1975和H1299细胞G_0_期/G_1_期占比分别为29.81%、49.27%，H1975DR和H1299DR细胞占比增加，分别为43.12%、59.02%。放射后72 h，细胞周期分布出现一定的恢复，总体趋势仍大致相同，提示放射后细胞周期的变化可能是H1975DR、H1299DR放射抵抗性增强的原因。

### 2.5 肺腺癌辐射耐受细胞株迁移及侵袭能力增强

肿瘤细胞放射抵抗的发生不仅与细胞抵抗凋亡、细胞周期再分布及DNA损伤修复增强相关，肺癌放射抵抗细胞往往具备更强的迁移及侵袭能力。在本研究中，我们采用细胞划痕对比亲本株及辐射耐受株的横向迁移能力。结果如图5A、图5B所示，H1975DR及H1299DR细胞在低血清培养基中划痕愈合速度快于H1975及H1299细胞，分别在24 h及30 h具有显著差异（P<0.05），提示辐射耐受株具有较强的迁移能力。同时，我们采用Matrigel基质胶模拟体内细胞外基质的功能和结构，在小室中检测细胞的侵袭能力，观察并统计上室细胞通过聚碳酸酯膜进入小室下室的细胞数目。结果如图5C、图5D所示，与亲本株H1975及H1299相比，辐射耐受株H1975DR、H1299DR在相同条件下细胞侵袭数目较多，结果表明，辐射耐受株H1975DR、H1299DR侵袭能力增强（P<0.01）。

**图5:**
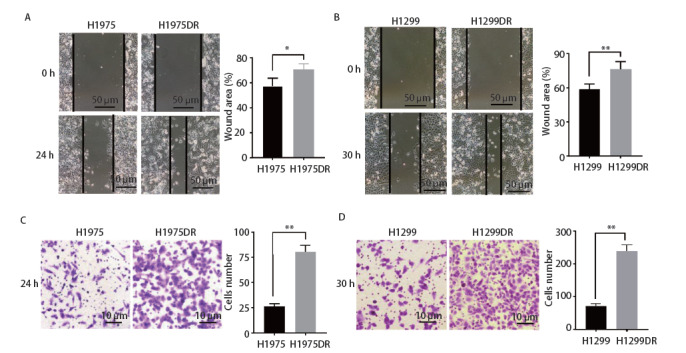
肺腺癌辐射耐受细胞株迁移及侵袭能力增强。 A、B：细胞划痕实验检测H1975/H1975DR和H1299/H1299DR细胞迁移能力的变化。右侧为细胞横向迁移面积统计图。C、D：Transwell细胞侵袭实验表明H1975DR和H1299DR细胞的侵袭能力增强。右侧为侵袭后细胞数目统计图。*P<0.05；**P<0.01。

### 2.6 肺腺癌辐射耐受细胞株DNA损伤修复蛋白表达的变化

基于肺腺癌放射抵抗细胞株较强的DNA损伤修复能力和放射抵抗表型，我们进一步探讨了DNA损伤修复因子的变化。IR诱导的DSB主要通过激活HR通路的ATM及NHEJ通路的DNA-PKcs，进而招募并磷酸化H2AX、RAD51、P53、53BP1等下游底物参与DNA损伤反应（DNA damage response, DDR）。我们采用Western blot免疫印迹法分析了HR通路中p-ATM、RAD51、p53和NHEJ通路中p-DNA-PKcs、KU70、KU80等分子的蛋白表达变化。结果如图6A、图B所示，单次剂量4 Gy照射24 h后可显著诱导p-ATM（Ser1981）和p-DNA-PKcs（Ser2056）的磷酸化，且H1975DR及H1299DR细胞株中p-ATM及p-DNA-PKcs磷酸化水平分别显著高于H1975及H1299细胞，提示肺腺癌放射抵抗细胞株具有更快更强响应DNA损伤修复的能力。这与辐射耐受株中更高的RAD51、p53及53BP1、γ-H2AX蛋白表达水平相一致。

**图6:**
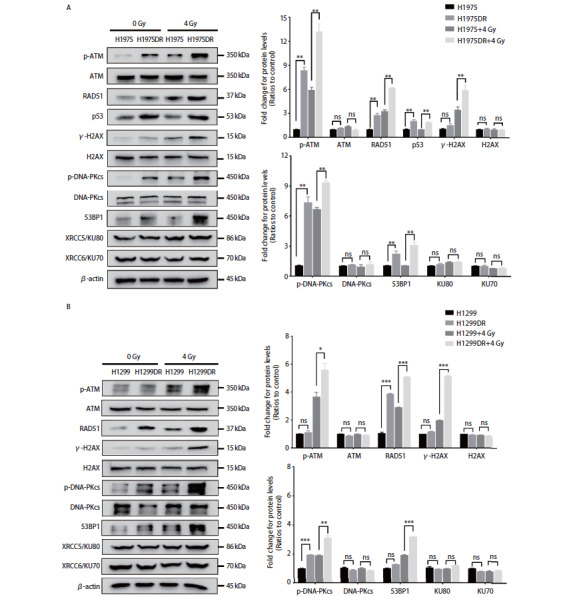
肺腺癌辐射耐受细胞株DNA损伤修复相关蛋白的改变。 A、B：采用Western blot检测H1975DR和H1299DR肺腺癌辐射耐受株中HR及NHEJ通路DNA损伤修复因子蛋白表达的变化。给予单次剂量4 Gy照射24 h后收集蛋白样本进行Western blot检测。右侧为蛋白相对表达量统计图。*P<0.05；**P<0.01；***P<0.001。

## 3 讨论

原发性及获得性放射抵抗是恶性肿瘤放射治疗失败的重要原因之一。近年来，国内外等多项研究^[11⇓⇓⇓⇓-16]^采用等剂量分割放疗的方法，成功构建了乳腺癌、结直肠癌、鼻咽癌、脑胶质瘤和宫颈癌等放射抵抗细胞系，并对其放疗耐受机制进行了不同程度的探索。在本研究中，我们挑选了肺腺癌细胞系H1299和H1975成功构建了放射抵抗细胞系H1299DR和H1975DR，并采用DNA损伤修复、抗凋亡、周期阻滞和迁移侵袭等表型实验对其放射抵抗性进行了对比验证，为肺癌患者放疗耐受的发生发展机制研究提供了体外细胞学模型。

肿瘤细胞放射抵抗的形成是射线选择性杀伤与细胞突变共同作用的结果。一方面，由于不同的细胞亚群及同一亚群细胞内环境的差异，射线选择性杀伤放射敏感性细胞株，使得放射相对抵抗性细胞株得以存活并不断增殖；另一方面，射线同时可诱导肿瘤细胞突变，使其具备放射抗拒表型，并遗传给子代细胞^[17]^。本研究选择H1975和H1299细胞株构建放射抵抗细胞系，在考虑到其病理类型的同时，还考虑到H1299为表皮生长因子受体（epidermal growth factor receptor, EGFR）野生型而H1975为EGFR 21L858R、20T790M突变型细胞系。已有研究^[18]^表明EGFR突变可增加NSCLC的放射敏感性，且EGFR突变的NSCLC患者因具有较高的应答率使其无进展生存期和总生存期均比野生型EGFR患者更长。因此，构建肺腺癌放射抵抗细胞模型，不仅对获得放射敏感或放射抵抗基因、筛选放疗获益人群有着重要的临床转化价值，也为优化EGFR靶向治疗联合放疗提供了细胞模型。

肿瘤细胞放射抵抗的产生是多因素、多通路及多机制共同调控的过程，IR诱导的DNA损伤修复、细胞周期阻滞、细胞凋亡、上皮间质转化（epithelial-mesenchymal transition, EMT）、肿瘤干细胞、致癌代谢和肿瘤微环境的改变等信号途径都参与放射抵抗的调节^[19]^。肿瘤细胞DNA损伤修复能力增强是发生放射抵抗的重要机制之一。射线诱导的DSB可迅速激活NHEJ和HR通路，在依赖于ATM的HR中，当监测到DSB时，MRE11-RAD50-NBS1（MRN）复合物迅速识别并结合DSB位点，在断口侧切开一个单链区，通过与ATM直接结合使其迁移到损伤部位并发生自磷酸化^[20]^。随后，ATM磷酸化大量下游底物，这些底物在DNA损伤位点积累并形成离散的foci，包括组蛋白H2AX、p53、RAD51等^[21]^。在NHEJ通路中，当DSB发生时，KU70/KU80异二聚体与DNA末端结合后的第一件事是募集DNA-PKcs，它是一个很大的蛋白，可以在两末端之间形成物理桥梁（physical bridge）来锚定KU复合物以完成后期修复。DSB可使断裂位点呈现“突出端”（overhangs）或糖基物残留，使得DNA连接酶IV（DNA ligase IV, Lig4）难以连接。此时，多核苷酸激酶（polynucleotide kinase, PKN）、核酸内切酶（ARTEMIS）和聚合酶在XRCC4和NHEJ1的帮助下，通过处理受损的DNA末端以促进Lig4的连接^[22]^。在本研究中，我们观察到H1975DR和H1299DR细胞的放射抵抗性显著增强，表现为H1975DR和H1299DR细胞的克隆形成率、存活分数及DNA损伤修复能力较亲本株H1975和H1299显著提高。且Western blot分析显示，在H1975DR及H1299DR辐射耐受株中，NHEJ修复通路的p-DNA-PKcs（Ser2056）、53BP1及HR修复通路的p-ATM（Ser1981）、RAD51相对蛋白表达量较H1975及H1299均有不同程度的增高，并具有X射线依赖性。已知IR诱导的DSB主要通过激活ATM和DNA-PKcs DNA损伤感受器，使其迁移到损伤部位并发生自磷酸化，从而磷酸化大量下游底物参与DDR，因此，H1975DR及H1299DR辐射耐受株可能通过同时调节NHEJ及HR DNA损伤修复途径增强了肺腺癌的放射抵抗性。

同时，IR诱导的细胞周期阻滞也是造成放射抵抗的原因之一。一般来说，G_2_期/M期细胞对射线最敏感，G_1_期细胞次之，S期细胞则最具放射抵抗性。如Liu等^[23]^研究表明放射后48 h，G_2_期/M期阻滞的细胞比例越高，细胞株的放射敏感性越低即放射抵抗性越强。一致的是，本研究发现在单次剂量6 Gy照射后48 h，辐射耐受株的细胞周期发生了再分布，表现为与H1975和H1299细胞株相比，H1975DR和H1299DR的G_2_期/M期比例均显著下调，并伴有放射相对抵抗的G_0_期/G_1_期比例的上调。在未照射时，H1975DR及H1299DR细胞周期分布与亲本株H1975及H1299细胞无显著差异，说明与H1975及H1299亲本株相比，H1975DR及H1299DR细胞株更具放射抗拒性。

此外，放射抵抗不仅与细胞周期阻滞、DNA修复因子表达量增高相关，也与放疗后的促血管生成效应有关。既往研究^[19]^表明，IR可通过诱导III型EMT以促进肿瘤迁移和侵袭，这与我们观察到的H1975DR及H1299DR细胞的迁移及侵袭能力增加相一致。有趣的是，我们通过显微镜观察发现辐射耐受细胞株具有明显的EMT形态学转变特性，表现为辐射耐受株的细胞间隙变大，纺锤样间质细胞增多，并形成多而长的伪足，其中H1299DR细胞株变化较为明显。因此，推测EMT可能介导H1975DR及H1299DR细胞放射抵抗的发生过程，但尚未对EMT相关标志物及转录因子和具体的作用机制展开研究。

本研究选用肺腺癌H1975和H1299细胞，经高能X射线分次照射，成功建立辐射耐受细胞株H1975DR、H1299DR，并对其细胞增殖、DNA损伤修复、凋亡、细胞周期、迁移和侵袭等多种放射抵抗表型和机制进行验证，多角度证明了该体外细胞模型的可靠性和可用性。基于此细胞模型平台，后期可以进一步利用RNA-SEQ和蛋白质谱的方法，筛选出放射敏感性差异表达基因和蛋白，这对于放射抵抗诊断标志物的确定、扩展肺癌放射抵抗的具体机制并最终在临床上实现以肺癌放射抵抗性分子为目标的靶向放疗或者联合治疗提供一定基础。
