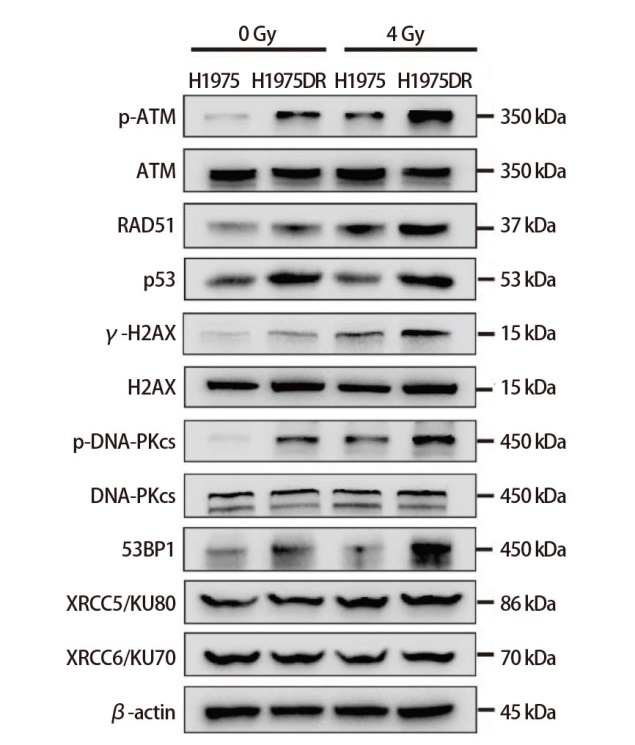# 更正声明

**DOI:** 10.3779/j.issn.1009-3419.2024.104.01

**Published:** 2024-10-20

本刊2023年第26卷第2期刊登的题为“人肺腺癌辐射耐受细胞株的建立及其放射抵抗机制探讨”[张静静, 马胜林, 吴琼, 等. 人肺腺癌辐射耐受细胞株的建立及其放射抵抗机制探讨. 中国肺癌杂志, 2023, 26(2): 93-104.]一文中：第102页，图6A中H2AX总蛋白条带图使用错误，正确结果如下图。出现上述错误系作者校对不仔细所致，特此更正。更正后的图片与原图的结论相同。因作者疏忽造成上述错误，对此深表歉意。

Erratum: Establishment of Human Lung Adenocarcinoma Radioresistant Cell Lines and the Mechanism of Radioresistance

Zhang JJ, Ma SL, Wu Q, et al.

Zhongguo Fei Ai Za Zhi, 2023, 26(2): 93-104.

Upon the initial publication of this article, error appeared on page 102, where the H2AX total protein band map in Figure 6A was incorrectly utilized. The corrected results are as follows:

**Figure:**